# Differences in cynomolgus macaque populations used for infectious disease research

**DOI:** 10.1002/ame2.70145

**Published:** 2026-02-09

**Authors:** Darcy Quist, Kimimuepigha Ebisine, Emma Kennedy, Stuart Dowall, Mike Dennis

**Affiliations:** ^1^ UK Health Security Agency (UKHSA) Salisbury UK

**Keywords:** animal models, communicable diseases, genetic variation, *Macaca fascicularis*, population genetics

## Abstract

Cynomolgus macaques, a species of Old World primate native to southeastern and eastern Asia and the island of Mauritius, are one of the most important nonhuman primate models for infectious disease. Although the closely related rhesus macaque is classified into subspecies based on geographic origin, no such subdivision exists for cynomolgus macaques, and they continue to be used interchangeably in infectious disease research, reducing the comparability of data produced from these studies. Research into the population genetics of cynomolgus macaques has found significant differences between macaques native to different areas, including their genetic diversity, with macaques from insular populations such as Mauritius and the Philippines exhibiting highly restricted heterozygosity compared to mainland populations native to Indonesia or Cambodia. In the context of infectious disease studies, research into pathogens, including Ebola virus, Crimean‐Congo hemorrhagic fever virus, and *Mycobacterium tuberculosis* have found differences in study outcomes, survival times, and immune cell responses between different populations of macaques. This review provides an overview of the differences between cynomolgus macaque populations in the context of genetic diversity, and in response to infection, and highlights the need for clear reporting of geographic origin of primates used in research. This will improve data comparison between studies and help to further refine this important animal model.

## Graphical abstract

Cynomolgus macaques, a species of Old World primate native to southeastern and eastern Asia and the island of Mauritius, are one of the most important nonhuman primate models for infectious disease. Research into the population genetics of cynomolgus macaques has found significant differences between macaques native to different areas, particularly with regard to their genetic diversity, immune cell responses, and disease susceptibility. This review provides an overview of the differences between cynomolgus macaque populations and highlights the need for clear reporting of geographic origin of primates used in research. Graphical abstract is produced using Biorender.com.

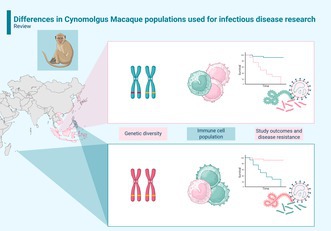

## 1 INTRODUCTION

Cynomolgus macaques (CMs; *Macaca fascicularis*), also known as crab‐eating or long‐tailed macaques, are a species of Old World primate with populations broadly distributed over southeastern and eastern Asia, as well as on the island of Mauritius off the east coast of Madagascar.^1^ Dispersal of *Macaca* populations northwards and southwards on the Asian continent around 2.31 million years ago (Mya)^2^ led to the speciation of cynomolgus and rhesus macaques (RMs; *Macaca mulatta*). Today, RMs and CMs are among the most important in vivo models for both communicable and noncommunicable disease research. Together, they account for approximately 95% of nonhuman primates (NHPs) used in research in the UK,^3^ and an estimated 96% of the primates imported to the USA.^4^ Although CMs are classified as the same species, regardless of their geographic origin, populations and individuals can differ dramatically, which could have consequences on the outcomes of studies using them in vaccine development or to model infectious disease.

For CMs used in research in the USA and the UK, the geographic origin of the animals is often difficult to determine from the literature alone. Many studies refer only to an “Asian” origin; others provide details only in Supporting Information, and some omit the information altogether. Some insight into the origins of CMs used in research can, however, be gained from trade data. As the trade of *Macaca* species is regulated under the Convention on International Trade in Endangered Species of Wild Fauna and Flora (CITES),^5^ data from the CITES Trade Database^6^ provide information on the countries exporting macaques to the UK and USA for biomedical research.

**FIGURE 1:**
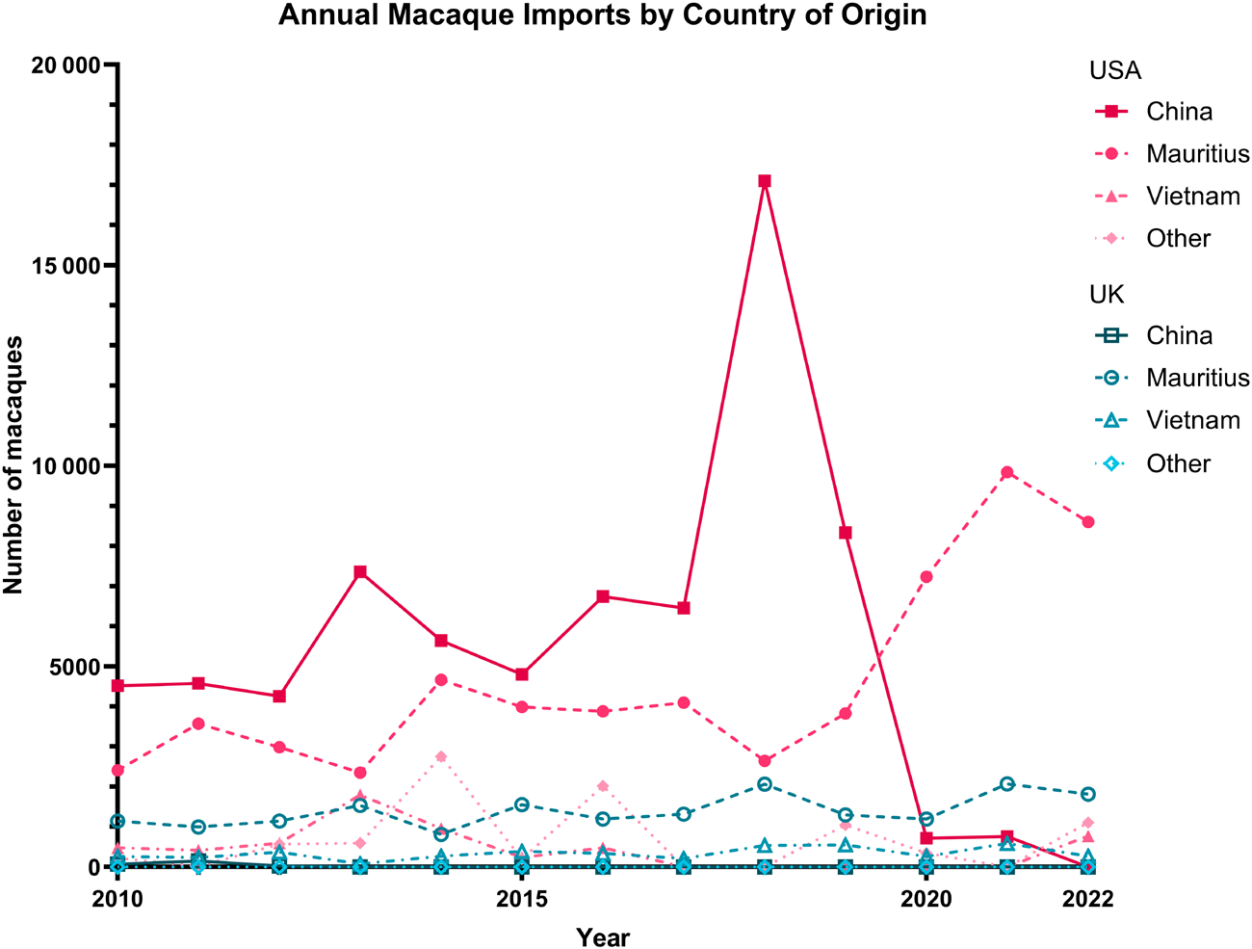
Graph showing the Convention on International Trade in Endangered Species of Wild Fauna and Flora (CITES)‐reported imports of cynomolgus macaques (*Macaca fascicularis*) into the USA and UK for biomedical research. The change in reported macaque imports into the USA (red) and the UK (blue) over a 12‐year period from 2010 to 2022 is shown based on country of origin: China, Mauritius, Vietnam, or “Other.” Countries that make up the “Other” countries are Cambodia (72%), the Philippines (17%), and Indonesia (11%). No imports into the UK from any of the “Other” countries were reported in CITES data over this period. The graph was produced using GraphPad Prism 10.

Although there are imperfections in CITES data,^7^ often due to reporting discrepancies and delays, year‐on‐year trends and the relative share of imports from each country can still be visualized (Figure 1), while acknowledging that reported and actual trade volumes may not align perfectly. Overall, China dominated macaque exports to the USA up until 2019–2020, when export restrictions were implemented to control the spread of COVID‐19. More recently, the majority of macaques imported into the USA have come from Mauritius, with smaller proportions from Vietnam, and countries such as Cambodia, the Philippines, and Indonesia. Although China accounted for most of the reported trade into the USA, Mauritius has remained the principal supplier to the UK throughout the past decade. The UK has shown less year‐on‐year fluctuation in imports, likely due to established macaque breeding colonies within the country, reducing reliance on foreign‐bred NHPs. These data reveal a research landscape often shaped by availability, rather than intent, where certain CM populations are used far more frequently than others. This imbalance may have implications for how results are compared and interpreted across studies.

Although in vivo studies using RMs tend to report whether the animals originated from China or India, including the source used from established breeding colonies, CMs continue to be treated as interchangeable in infectious disease research. Previous investigations into the impact of geographic origin of CMs used in biomedical research have been conducted across a range of disciplines.^8^, ^9^ In the context of drug safety evaluation, a 2015 study examined the pharmacokinetics and safety profiles of midazolam (a benzodiazepine) and efavirenz (an antiretroviral) in 60 CMs originating from Cambodia, Mauritius, and a group of mixed Asian/Indochinese origin supplied by a Chinese biotechnology company.^10^ In addition to differences in background observations, where Mauritian CMs displayed less variability and fewer spontaneous pathological findings, marked differences were reported in histopathological features among populations. Mauritian CMs exhibited a lower incidence and severity of lymphoid hyperplasia and reduced mononuclear cell infiltration in the stomach, kidneys, heart, and lungs. In contrast, Cambodian CMs showed a high incidence of seminiferous tubule dilation with associated epithelial degeneration, and both Cambodian and mixed Asian CMs demonstrated a greater degree of testicular immaturity than their Mauritian counterparts. Although none of these findings were considered detrimental to the use of any CM population in drug‐safety studies, the authors emphasized the importance of accounting for animal origin during study design.

Similar population‐dependent variation has also been reported in clinical chemistry and hematological parameters, further emphasizing the influence of genetic background on physiological baselines in CMs. A 2022 study compared routine hematology, coagulation, and clinical chemistry endpoints between Mauritian CMs and Asian CMs originating from Cambodia, China (from a Chinese breeding facility), and Vietnam.^11^ Mauritian macaques were found to have a lower mean corpuscular volume (a measure of red blood cell size) and a lower red blood cell count compared to Asian CMs, whereas macaques from Vietnam exhibited a shorter prothrombin time (a measure of coagulation) than those from Mauritius. Further differences were observed in serum biochemistry, with Asian macaques displaying lower gamma glutamyl transferase activity and lower concentrations of serum total protein and globulin, as well as higher cholesterol, than Mauritian CMs. Together, these findings highlight the physiological variation between regional populations of CMs; however, a gap in the literature remains regarding how such differences influence in vivo disease pathogenesis. In this review, the differences between CM populations and their suitability as in vivo models for selected infectious diseases will be evaluated.

## 2 MAIN

### 2.1 Dispersal and population genetics

**FIGURE 2:**
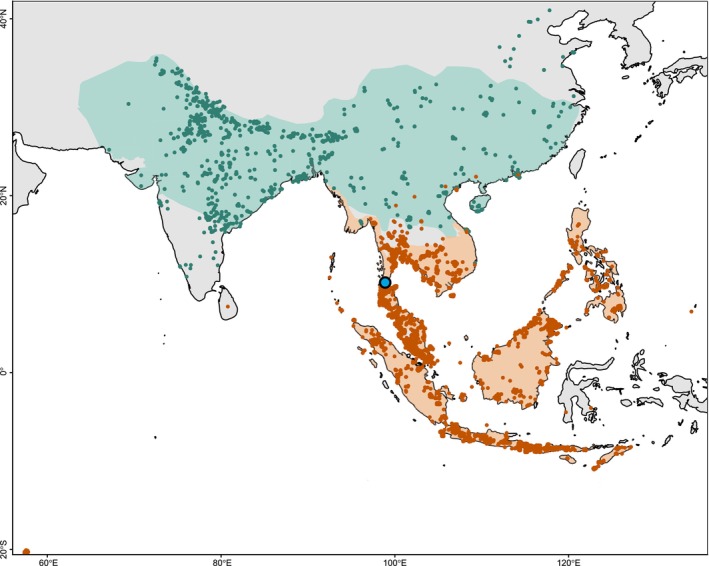
Geographic distribution of rhesus macaques (*Macaca mulatta*) and cynomolgus macaques (*Macaca fascicularis*) in South and Southeast Asia. Orange shading indicates the native range of rhesus macaques, whereas teal shading represents the native range of cynomolgus macaques. Points correspond to individual occurrence records obtained from the Global Biodiversity Information Facility (GBIF).^14^, ^15^ Shaded areas align with species' distributions according to the International Union for Conservation of Nature (IUCN).^16^, ^17^ The map illustrates the broad dispersal of both species, including regional populations of cynomolgus macaques across the Indochinese Peninsula, the Malay Peninsula, the Sunda Shelf islands, and the introduced population on Mauritius. The Isthmus of Kra is marked in blue at the center of the map. The map was generated using RStudio (version 4.4.1).

Following their divergence, and likely driven by interspecific competition both between the two species and with other members of the *Macaca* genus, RMs and CMs dispersed northwards and southwards, respectively, occupying extensive areas across South and Southeast Asia. Today, RMs have the widest native range of any NHP, inhabiting regions of 11 countries, including India, China, Afghanistan, and Vietnam.^12^ CMs, in contrast, occupy much of mainland Southeast Asia as well as numerous shallow‐ and deep‐water islands on and beyond the Sunda Shelf, and an introduced population on the island of Mauritius, east of Madagascar.^13^ Figure 2 illustrates the native ranges of both RMs (orange) and CMs (teal), with points representing individual occurrence records from the Global Biodiversity Information Facility (GBIF),^14^, ^15^ and shaded areas corresponding to species distributions according to the International Union for Conservation of Nature (IUCN).^16^, ^17^ Mitochondrial and nuclear genetic analyses consistently identify several regional lineages corresponding broadly to Indochinese, Indonesian, and Philippine populations, in addition to the derived Mauritian population, reflecting both historical isolation and variable gene flow among island and mainland lineages.^18^ The dispersal history and geography of each regional CM population have shaped their population genetics and, consequently, their responses to infectious disease, with each population possessing distinct attributes, advantages, and limitations.

Modern trade and large‐scale breeding practices have blurred the geographic boundaries that once defined these populations, complicating efforts to identify the true genetic origin of research animals. Although trade data provide valuable insight into the flow of macaques for research, the country of export does not necessarily correspond to the animals' genetic origin. Many CMs exported from China, for example, are bred in commercial farms using breeding stock imported from countries within the species' native range, including Indonesia, Vietnam, and Cambodia.^19^ Studies using these CMs often cite the animals as being of “Chinese origin,” despite the fact that *M. fascicularis* is not indigenous to China. Consequently, these “Chinese” CMs have been the subject of research investigating their ancestry and the genetic composition of breeding populations.

A 2017 study constructed a panel of 96 single nucleotide polymorphisms (SNPs) to determine both the geographic origin and the extent of RM ancestry in 400 CM DNA samples from 10 Chinese breeding farms.^20^ Of the 391 samples included in downstream analyses, almost all macaques were determined to be of exclusively Indochinese ancestry, with the other 4 samples originating from the same farm. These findings suggest that the majority of so‐called “Chinese” CMs are in fact of Indochinese origin. The combined population displayed low fixation index (F_ST_) and inbreeding coefficient (F_IS_) values (0.007 and 0.047, respectively), indicating that despite minimal inbreeding within the farms (low F_IS_), the population is remarkably genetically homogenous (low F_ST_). Although all 391 samples revealed some evidence of RM introgression, only 38% exhibited levels above the mean of 17%. This pattern is not due to ongoing hybridization within Chinese breeding farms, of which few, if any, macaques were first‐generation hybrids, but rather reflects ancient introgression from Chinese RMs to Indochinese CMs over thousands of years in wild populations north of the Isthmus of Kra (the location of which is marked by a blue point in Figure 2).^21^, ^22^ This admixture contributes to the high genetic diversity observed in Indochinese macaques, but any RM‐derived alleles present in Indochinese CMs may also influence susceptibility to certain pathogens. In the case of infection with *Plasmodium cynomolgi*, a malaria‐causing eukaryotic parasite, RMs appear more prone to infection and typically experience more severe disease than CMs.^23^ Consistent with this, the study described above found a strong association between the extent of RM introgression in Indochinese CMs and the likelihood of *P. cynomolgi* infection.

The population genetics and altered disease susceptibility of these animals have important implications for research involving Indochinese or China‐sourced CMs. Greater genetic diversity may improve the predictive value of disease models by more closely mirroring human population variability; however, it also introduces potential confounding effects due to interindividual differences in immune responses. Furthermore, it is important that authors accurately report the origin of their Chinese CMs. Although many studies (including examples later in this review) describe their animals as Chinese, referring to the location of the breeding facility, greater care should be taken in future to specify their true geographic origin or, if unknown, to refer to them more accurately as Indochinese.

Though the majority of CMs in the wild are found across Mainland Southeast Asia, they were introduced into Mauritius around 500 years ago and are now an established (and invasive^24^) species on the island. Analysis of the mitochondrial and Y‐chromosomal DNA of these Mauritius CMs and comparison with macaques from insular and continental Southeast Asia^25^, ^26^, ^27^ has revealed that a mixed origin for the Mauritius population is likely, with DNA shared with Javan, Sumatran, and Indochinese macaque lineages.

Despite their relatively recent introduction to the island, the Mauritian population has been shown via genetic variance (F_ST_) analysis to be the most divergent from the assumed source population when compared to macaque populations from four other locations across Southeast Asia.^28^ The Mauritian population also exhibits a low level of genetic diversity, with lower levels of heterozygosity and estimated inbreeding coefficients (F_IS_/F_IT_) significantly higher than in other RM and CM populations,^18^, ^27^, ^29^, ^30^, ^31^ indicating a higher level of inbreeding.

The genetic homogeneity of the Mauritian macaques is likely the result of a strong founder effect followed by genetic drift.^32^ The founder effect occurs when a small number of individuals establish a new, often geographically isolated population that is not genetically representative of the source population.^33^ Due to the small population size, random fluctuations in allele frequencies (genetic drift) and inbreeding further reduce genetic variation over time. In the case of Mauritius, a small number of macaques were introduced anthropogenically, presumably as escaped pets from Portuguese ships.^32^ Allele frequency data and population demographics have been used to estimate the number of original founders to be as low as 10–15 individuals,^30^, ^31^ with a male‐biased sex ratio resulting in the significant lack of polymorphism seen in the mitochondrial DNA (mtDNA) of Mauritian CMs compared to other populations.^25^ This genetic uniformity has implications for their use in infectious disease research. Although the reduced interindividual variability offers advantages in terms of experimental reproducibility and statistical power, potentially reducing the number of macaques required per study, the relevance of such findings may be limited when applied to genetically diverse human populations.

The macaques of Mauritius represent a recent introduction; however, another, much older insular population of CMs is found in the Philippines. Two distinct subspecies of *M. fascicularis* occur across the Philippine islands, both used as animal models in biomedical research and both the result of separate introductions. *M. f. fascicularis*, the nominotypical CM subspecies found throughout mainland and Southeast Asia, inhabits the south‐central islands and exhibits a light‐to‐medium pelage resembling that of other populations, whereas *M. f. philippensis*, which has a distinctive dark dorsal pelage, occupies most of the remaining islands.^13^ Although the Indochinese Peninsula, the Malay Peninsula, Sumatra, Borneo, and Java were once connected to mainland Southeast Asia on the Sunda Shelf, allowing the dispersal of CMs across this area, the Philippines are separated from the Sunda Shelf by steep underwater gradients and likely originated as de novo oceanic islands.^34^ This separation limited opportunities for colonization to periods of glacial maxima, when sea levels dropped sufficiently to narrow the water gap and permit occasional overwater dispersal from Borneo via sea rafting. The first introduction of *M. fascicularis* to the Philippines likely occurred during the penultimate glacial maximum, with approximately 160 000 years of isolation giving rise to the darker, differentiated subspecies *M. f. philippensis*. In contrast, the second introduction probably took place during the most recent glacial maximum around 18 000 years ago, and this population has not diverged sufficiently to be considered distinct from *M. f. fascicularis*, retaining the typical lighter pelage of its mainland counterparts.^35^

Despite the existence of two subspecies on the Philippine islands, the specific subspecies or region of origin of CMs used in biomedical research is rarely reported. Although these macaques are not as geographically separated from their Indonesian homeland as the Mauritian population, they nevertheless show marked differences in genetic composition and diversity. A 2007 study analyzed mtDNA variation among regional macaque populations using samples from 459 CMs and 594 individuals from three other *Macaca* species, including *M. mulatta*.^18^ Philippine *M. f. fascicularis* exhibited the second lowest within‐population nucleotide diversity (*π* = 0.018) of any other regional macaque population surpassed only by Mauritian CMs (*π* = 0.0008). Moreover, both Philippine and Mauritian macaques were the most divergent from Indochinese CMs (*π* = 0.080 and *π* = 0.090, respectively), with the genetic distance between Philippine and Indochinese populations exceeding that observed between Chinese and Indian RMs (*π* = 0.076). A separate study comparing *M. f. fascicularis* and *M. f. philippensis* found that the latter exhibited even lower mtDNA diversity (*π* = 0.0005).^36^ The two Philippine populations were also more distinct from each other (*π* = 0.039) than either was from Sarawak (*M. f. philippensis π* = 0.033, and *M. f. fascicularis π* = 0.030), supporting the hypothesis that both lineages arrived independently from Borneo. Collectively, these findings highlight both the genetic divergence of Philippine CMs from other Southeast Asian populations and the need for caution when treating them as interchangeable in infectious disease research. Their limited genetic diversity offers similar advantages and drawbacks to Mauritian CMs when used as in vivo models.

In contrast, CMs from other regions of Southeast Asia exhibit substantially higher genetic diversity. Analyses of the genome, including studies of the major histocompatibility complex (MHC),^37^ indicate that populations from Indonesia, Indochina, and Cambodia are not only more diverse than their Mauritian counterparts^18^, ^28^, ^38^ but also genetically distinct from each other. Some populations, such as those from Indochina and the Philippines, differ from each other to an extent comparable to the divergence between Chinese and Indian RMs,^18^, ^39^, ^40^ two “subspecies” that are as genetically distant from each other as they are from other *Macaca* species.^41^, ^42^ Although most of these studies focused on wild‐caught CMs, research by Mee et al.^43^ and Mitchell et al.^44^ has shown that captive Indonesian and Mauritian macaques maintain the population genetics of their wild counterparts, reflecting the restricted MHC polymorphism in Mauritius macaques and the extensive diversity present in Indonesian populations.

In addition to studies on the MHC, other genetic factors exert effects. Genes encoding killer‐cell immunoglobulin‐like receptor (KIR), expressed on natural killer (NK) cells and subsets of T lymphocytes,^45^ have also been studied in CM populations where multiple alleles have been identified.^46^, ^47^, ^48^ When comparisons were made between different origins of CMs, it was observed that those from the Malaysian mainland had, on average, one additional inhibitory KIR receptor compared to those that inhabit the Indonesian/Malaysian islands.^49^ In the RM simian immunodeficiency virus (SIV) infection model, the kinetics of plasma viral loads are correlated with different inhibitory and activating KIR genes^50^; therefore, for CM it is also expected that disease pathogenesis may be influenced by KIR expression. Similar to KIR expression, cytochrome P450 gene families have also been demonstrated to vary in CMs.^51^, ^52^, ^53^ As these families of endogenous and exogenous substrate‐metabolizing enzymes play key roles in drug metabolism, they can influence the suitability of CMs for such studies.^54^, ^55^ When animals sourced from breeding units in three distinct geographical regions (Cambodia, China, and Indonesia) were investigated, expression levels of 14 cytochromes P450 did not significantly differ (>2.5‐fold) at this population level.^56^ Collectively, the marked genetic differences among CM populations, including variation in MHC, KIR, and cytochrome P450 loci, suggest that the geographic origin of macaques may influence their physiological and immunological responses, directly impacting outcomes in infectious disease research.

### 2.2 Differences in infectious disease study outcomes

#### 2.2.1 Ebola

The Filoviridae is a family of RNA viruses that includes the genus *Orthoebolaviruses*, which contains Ebola virus (EBOV), the causative agent of the often severe Ebola hemorrhagic fever.^57^ EBOV is responsible for regular outbreaks of viral hemorrhagic fever, including the largest in West Africa during 2014–2016, which resulted in the deaths of over 10 000 people.^58^ The CM is a reliable NHP model for EBOV infection, with pathology closely mirroring human disease and the potential for fatal outcomes enabling meaningful assessment of vaccine efficacy.^59^ Various NHP species have been utilized to model EBOV infection, with RMs and CMs being the most commonly used. For vaccine development, CMs are often preferred because they exhibit a more compact disease course and a more consistent pattern of disease progression compared to RMs, which are more frequently employed for postexposure therapeutic studies.^60^, ^61^ EBOV studies have used CMs from several geographic origins, including Mauritian,^62^ Chinese,^63^, ^64^, ^65^ and Vietnamese populations.^66^, ^67^, ^68^ In many other studies, however, the origin of macaques is not reported or appears only in the Supporting Information methods. This omission complicates cross‐study comparisons, as population‐specific differences in immunological gene systems and polymorphisms may influence EBOV susceptibility and pathogenesis.

In a 2021 study, Niemuth et al.^69^ investigated outcomes in 122 CMs infected with EBOV. The researchers analyzed a large database of sham‐vaccinated control CMs across 33 EBOV vaccine studies, with EBOV doses ranging from 0.1 to 100 000 plaque‐forming units administered via various exposure routes. Variables, including time to death and viral load, were compared against factors such as macaque origin (“Asian” or “Mauritius”), sex, and age. In the sham‐vaccinated control group, viral loads appeared in the blood within 3–5 days, leading to rapid disease progression and euthanasia or death within 5–12 days postinfection. Reduced activity and responsiveness were typically observed 4–6 days after infection, preceding death/euthanasia by approximately 1 day. Disease progressed rapidly, as evidenced by changes in body temperature and blood profiles, particularly those indicative of coagulation disruption. The analysis found that demographic variables, control article, and euthanasia criteria were not statistically significant predictors of time to death. When comparing macaque origin, a log‐rank comparison found that Mauritian CMs had a significantly shorter (*p* = 0.0228) time to death than Asian CMs. Although this result was not statistically significant in the more robust Cox PH‐frailty model, the hazard ratio of 1.45 suggested a possible higher risk in Mauritian CMs. Furthermore, although the 95% confidence intervals (CIs) were wide and included 1.0, they were weighted toward a hazard ratio >1 (0.63–3.36), supporting the hypothesis that macaque origin may influence disease outcome.

NHP models have also been used for other viruses in the Filoviridae family, including Marburg virus (MARV). In one study,^70^ the authors acknowledged that macaque origin was an uncontrolled variable that could have contributed to differences in median survival time after MARV exposure when comparing their data to existing literature.

Although there is a gap in EBOV literature directly comparing disease outcomes between CMs of different origin, the possibility that origin affects survival time has been repeatedly suggested. This variability was summarized in the review by Nakayama and Saijo^71^ who noted that species origin may influence EBOV disease pathology and progression.

#### 2.2.2 Crimean‐Congo hemorrhagic fever

Crimean‐Congo hemorrhagic fever (CCHF) virus is spread by *Hyalomma* ticks and is the most widespread viral tick‐borne infection worldwide, endemic in areas south of the 50th parallel north, including countries in Africa, the Balkans, the Middle East, and Asia.^72^ The reported case fatality rate varies but is generally accepted to be around 5%, and as cases are generally sporadic, clinical trials would likely be evaluated under the animal rule.^73^ The CM model represents an important advancement in this field, as previous infection studies in other primates, including African green monkeys and baboons, have proved to be unsuccessful.^74^

Although CCHF has been the recognized cause of outbreaks since 1944, and similar tick‐borne hemorrhagic fevers have been described since at least the 12th century,^75^ there have not been a large number of infection studies carried out in CMs. This is in part due to the recent characterization of the model,^74^ as well as the requirement for CCHFV research to be carried out under Containment Level 4 (CL4) or Biosafety Level 4 (BSL‐4) conditions,^76^ restricting work to a limited number of facilities capable of housing NHPs infected with hazard group 4 pathogens.

The use of CMs as a model for CCHFV studies is relatively new, with the first study published in 2018.^77^ As a result, there is less variation in the origin of macaques used. In studies where origin was reported, most macaques used were described as “Chinese.”^78^ In 2020, Cross et al.^79^ infected both Mauritius and “Chinese” CMs to discern a difference in CCHF disease course, hypothesizing that the Mauritius CMs would replicate human disease more closely. Although the researchers reported no significant differences, the cohort was very small (*n* = 6 animals) and due to study limitations regarding virus stock, macaques were further split into groups of *n* = 1 and *n* = 2, with uneven sexes in comparative groups.

Discrepancies between outcomes between institutions have been reported, with genetic differences between NHPs being suggested as a cause^74^; therefore, refinement of the CM CCHF model is essential for future vaccine evaluation. Further research, especially a direct comparison study with large group sizes of equal sexes, is needed to properly address whether clinical outcomes of CCHFV infection differ in CMs of different origin.

#### 2.2.3 Tuberculosis

Tuberculosis (TB), caused by the *Mycobacterium tuberculosis* bacteria, is a disease with a considerable burden worldwide. TB is responsible for the deaths of over a million people annually and is the leading cause of adult death from infectious disease globally.^80^ *M. tuberculosis* infection in CMs closely resembles human disease and has been a useful model for over two decades,^81^ with low‐dose inoculation resulting in both active–chronic and latent infections in these NHPs.^82^

Although differences in infection and vaccine efficacy between CMs and RMs were reported as early as 2001,^83^ investigations into the differences among CMs of distinct geographic origins did not appear until more recently. The first TB dose‐ranging study in Mauritius CMs was carried out in 2017 by Sharpe et al.,^84^ and the authors compared their outcomes with the clinical signs and disease features exhibited in both RMs and Chinese CMs. Mauritius CMs challenged via aerosol were less able to control infection than their Chinese counterparts, and clinical signs of altered respiratory rate and coughing, absent in Chinese CMs, occurred in all Mauritius CMs exposed to low, medium, and high doses of TB.

To investigate some of the factors underpinning these differences, a 2019 study aimed to assess the susceptibility of macaque populations to TB and compare monocyte‐to‐lymphocyte ratios both before and after infection using data from previous studies.^85^ The monocyte‐to‐lymphocyte ratio (M:L) in peripheral blood can be a predictor of the severity of TB infection in humans, with a higher M:L associated with active TB infection, and a greater proportion of lymphocytes indicative of latent infection.^86^ Researchers found that Mauritius CMs all met humane endpoints within 12 weeks; in contrast, Chinese CMs were able to control TB infection in most cases, with clinical endpoints only reached in macaques given the highest doses of TB.^85^ In measurements taken both pre‐ and post‐TB infection, M:L ratio was found to be significantly higher in Mauritius CMs than Chinese CMs, and Mauritius CMs controlling the disease were still found to have a significantly higher M:L than their Chinese counterparts. Furthermore, the M:L differences in Chinese CMs and Mauritius CMs were much larger than those between Mauritius CMs and RMs, despite being separate species. A further study in 2021, comparing blood immune cell composition in Mauritius, Chinese, and Indonesian CM populations, found additional significant differences between the geographical origin of these macaques. Cell populations of monocytes and eosinophils were higher in Indonesian CMs than Chinese or Mauritius CMs, but the numbers of neutrophils in Indonesian CM blood samples were significantly lower. This resulted in a higher M:L ratio in Indonesian and Mauritius CMs compared to Chinese CMs, but a lower neutrophil‐to‐lymphocyte ratio in Indonesian CMs than in either of the others.^87^ Together, these studies further contribute to the growing evidence for disparity in TB disease outcome and immune response in macaques of varied geographical origin.

## 3 CONCLUSIONS AND FUTURE DIRECTION

Despite the generalization that all CMs are the same species, especially in their use as a model for infectious diseases, many differences exist in their population genetics and genetic histories, as well as in disease outcomes, survival, and immune responses. Through the examples described in this article, a possible trend of reduced immune resilience in CMs from Mauritius has emerged in both viral and bacterial infections, with CMs from areas such as Indonesia having a predisposition to being more resilient to severe disease.

As macaque studies are often restricted to small group sizes, it is essential for study designers to be well informed when selecting the appropriate CM populations to meet the aims of their research. Choosing animals of known geographic origin can substantially enhance the translational relevance of a study by more accurately reflecting variation observed in human populations. Researchers should therefore make it standard practice to report the origin of CMs used in infectious disease studies to facilitate meaningful cross‐study comparisons. For macaques where the geographic origin is unknown, molecular tools such as mtDNA sequencing^18^, ^88^ or SNP‐based identification panels, such as the 48 ancestry‐informative marker assay described by Zhang et al.,^20^ provide a rapid, reliable, and cost‐effective means of determining ancestry. If the origin of all captive CMs was determined in this way, comparison between studies across multiple biomedical fields would become more relevant and more informative.

Additionally, there are increasing efforts for the inclusion of both sexes, where practical, when conducting preclinical modeling studies.^89^ Although outside the focus of this review, consideration of the impact of introducing other variables is required in study design when using CMs. For example, levels of mtDNA may exert differences between the sexes^90^ and have been shown to have an influence on biological outcomes such as aging in macaques.^91^ Likewise, sex‐biased genes,^92^ such as Y‐DNA on the male chromosome, have also been shown to exert effects in other animal species.^93^ Furthermore, due to their unique genetic histories, CMs from different lineages exhibit markedly different levels of diversity in their mtDNA^25^ and Y‐DNA.^94^ Mauritian macaques, for instance, are thought to have originated from a founding population of only a few females, resulting in exceptionally low mtDNA nucleotide diversity. In one study analyzing an 1800 base pair mtDNA fragment, only a single polymorphic site was identified among 19 Mauritian CMs, and both mitochondrial haplotypes present in the population were derived from the same Indonesian haplotype.^32^ Consideration of these factors is of particular importance where macaque models are required as preclinical models for newly emerging health threats where a rapid assessment of pathogenesis and supporting data for regulatory submission of vaccines or therapies is required.

Although an in‐depth analysis into the factors underpinning the differences in these populations is beyond the scope of this paper, future research into the direct impact of the restricted MHC polymorphism in Mauritian CMs, or via meta‐analysis of clinical data, is urgently needed. Furthermore, the use of CMs in research is not limited to infectious diseases. The majority of CMs in laboratories in the UK are used for regulatory procedures, including toxicity and safety testing, pharmacodynamics, and repeat‐dose toxicity.^3^ As the outcomes of these studies directly inform safety guidelines in the licensing of drugs and pharmaceuticals, further research into the differences in CM populations is essential to better inform study design and facilitate accurate comparisons of data between studies and institutions.

## AUTHOR CONTRIBUTIONS

**Darcy Quist:** Investigation; visualization; writing – original draft; writing – review and editing. **Kimimuepigha Ebisine:** Investigation; writing – review and editing. **Emma Kennedy:** Conceptualization; project administration; supervision. **Stuart Dowall:** Conceptualization; funding acquisition; project administration; supervision; writing – review and editing. **Mike Dennis:** Writing – review and editing.

## FUNDING INFORMATION

This research received no external funding.

## CONFLICT OF INTEREST STATEMENT

The authors have no conflicts of interest to declare.

## ETHICS STATEMENT

Due to being a review article, no ethical approvals were required.

## Data Availability

No new data were created or analyzed in this study. Data sharing is not applicable to this article.
